# First experiences using transurethral ultrasound ablation (TULSA) as a promising focal approach to treat localized prostate cancer: a monocentric study

**DOI:** 10.1186/s12894-023-01306-6

**Published:** 2023-08-29

**Authors:** Inga Peters, Bennet Hensen, Julian Glandorf, Marcel Gutberlet, Martha Dohna, Steffen Struckmann, Markus Antonius Kuczyk, Frank Wacker, Susanne Hellms

**Affiliations:** 1 Clinic for Urology, Krankenhaus Nordwest, Steinbacher Hohl 2-26, 60488 Frankfurt, Germany; 2 Department of Urology and Urologic Oncology, Hannover Medical School, Carl- Neuberg Str. 1, 30625 Hannover, Germany; 3 Institute for Diagnostic and Interventional Radiology, Hannover Medical School, Carl- Neuberg Str. 1, 30625 Hannover, Germany

**Keywords:** Focal therapy, Prostate cancer, Multiparametric MRI, Prostate-specific antigen, Thermometry, Transurethral ultrasound ablation

## Abstract

**Purpose:**

To share our experience using transurethral ultrasound ablation (TULSA) treatment for focal therapy of localized prostate cancer (PCa).

**Materials and methods:**

Between 10/2019 and 06/2021 TULSA treatment for localized PCa was performed in 22 men (mean age: 67 ± 7 years, mean initial PSA: 6.8 ± 2.1 ng/ml, ISUP 1 in n = 6, ISUP 2 in n = 14 and 2 patients with recurrence after previous radiotherapy). Patients were selected by an interdisciplinary team, taking clinical parameters, histopathology from targeted or systematic biopsies, mpMRI and patients preferences into consideration. Patients were thoroughly informed about alternative treatment options and that TULSA is an individual treatment approach. High-intensity ultrasound was applied using an ablation device placed in the prostatic urethra. Heat-development within the prostatic tissue was monitored using MR-thermometry. Challenges during the ablation procedure and follow-up of oncologic and functional outcome of at least 12 months after TULSA treatment were documented.

**Results:**

No major adverse events were documented. In the 12 month follow-up period, no significant changes of urinary continence, irritative/obstructive voiding symptoms, bowel irritation or hormonal symptoms were reported according to the Expanded Prostate Cancer Index Composite (EPIC) score. Erectile function was significantly impaired 3–6 months (p < 0.01) and 9–12 months (p < 0.05) after TULSA. PSA values significantly decreased after therapy (2.1 ± 1.8 vs. 6.8 ± 2.1 ng/ml, p < 0.001). PCa recurrence rate was 23% (5/22 patients).

**Conclusion:**

Establishment of TULSA in clinical routine was unproblematic, short-term outcome seems to be encouraging. The risk of erectile function impairment requires elaborate information of the patient.

## Introduction

Treatment options for localized prostate cancer (PCa) range from radical prostatectomy, radiation therapy, and focal therapy to active surveillance. Careful patient selection is the essential task for appropriate oncologic treatment while preserving good life quality.

Different techniques for focal ablative therapy are currently available such as cryotherapy, high-intensity focused ultrasound (HIFU) or vascular-targeted photodynamic therapy (TOOKAD). Focal therapies have the potential to provide proper oncologic treatment while preserving urinary continence and erectile function due to a nerve-sparing application [1]. Multiple options for the treatment of PCa allow for personalized and flexible therapies to each individual patient.

A recent multicenter, phase 2b study reports that 24-months biopsy outcomes show that focused ultrasound focal therapy with Magnetic Resonance Imaging (MRI) guidance is safe and effectively treats grade group 2 or 3 PCa [2]. A novel focal approach to treat PCa is transurethral ultrasound ablation (TULSA). For TULSA, a rotating ultrasound probe is placed in the prostatic urethra to apply heat to the full prostatic tissue. Real-time MRI-thermometry assures monitoring of heat development during the entire procedure. Recent studies could show effective tissue ablation and PSA reduction as well as low rates of toxicity and residual disease after whole gland ablation applying whole-gland TULSA in men with localized prostate cancer [3, 4]. Accurate and safe ablation of PCa has also been reported for lesion-targeted, focal TULSA [5, 6] and for symptomatic locally advanced prostate cancer in a study performing palliative TULSA treatment in 10 men [7].

With this study, we want to share our experience with the TULSA-PRO device (Profound Medical Corp., Mississauga, Canada). We aim to report perioperative difficulties as well as oncologic and functional follow-up.

## Materials and methods

### Patients

22 men received TULSA treatment of localized PCa between 10/2019 and 06/2021. 6/22 TULSA treatments were whole-gland ablations and 16/22 were focal ablations (25–60% of the prostate tissue). Patients’ mean age was 67 ± 7 years. Mean initial PSA value was 6.8 ± 2.1 ng/ml. Gleason scores (GS) before treatment were 3 + 4 = 7a in 14 patients, 3 + 3 = 6 in 6 patients, and 2 patients were treated as salvage procedure due to recurrence after previous radiotherapy. Prior to treatment none of the patients received androgen-deprivation therapy. Patient characteristics are summarized in Table 1.

**Table 1:** Patient characteristics

ID	Initial Gleason score	ISUP/ D’Amico	Ablation mode	Initial PSA ng/ml	Follow-up PSA ng/ml	Follow-up MRI-PI-RADS	Follow-up histopathology from MRI/ultrasound fusion-guided biopsy, infield (i) / outfield (o)	Specific characteristic
1	7a	2/ir	focal	8	4.22	4	7a (i + o)	artifact during TULSA
2	7a	2/ir	focal	7.1	4.1	4	7a (o)	
3	7a	2/ir	focal	5	2.9	2	no malignancy	
4	7a	2/ir	focal	10.3	1.59	2	no malignancy	
5	6	1/lr	whole-gland	6.2	0.01	2	NA	
6	7a	2/ir	whole-gland	7.05	0.15	2	no malignancy	
7	7a	2/ir	focal	9.15	0.75	2	no malignancy	
8	7a	2/ir	focal	8.1	4.6	2	no malignancy	
9	6	1/lr	whole-gland	9.5	0.34	2	no malignancy	
10	7a	2/ir	focal	5.5	5.5	2	NA	
11	7a	2/ir	focal	3.46	4.23	4	7a (i + o)	
12	7a	2/ir	focal	7.3	0.43	2	no malignancy	
13	6	1/lr	focal	6.4	3.25	2	NA	
14	7a	2/ir	whole-gland	8.8	0.71	2	NA	
15	7a	2/ir	focal	9.1	2.37	2	no malignancy	
16	7a	2/ir	focal	5.5	1.4	2	NA	
17	7a	2/ir	focal	7.13	1.26	2	no malignancy	
18	6	1/lr	focal	5		NA	NA	Lost to follow-up
19	7a	2/ir	focal	2.6	1.78	3	7b (o)	
20	7a	2/ir	focal	5.33	3.05	4	NA	Lymph-node metastasis diagnosed with PSMA-PET
Salvage treatment after previous radiotherapy								
21	No GS after radiotherapy		whole-gland, salvage	4.5	0.08	3	no malignancy	Later diagnosed with bone metastasis
22	No GS after radiotherapy		whole-gland, salvage	9.53	0.06	NA	NA	Patient refused MRI and Biopsy at PSA 0.1 ng/ml

PSA = prostate specific antigen, NA = not available, i = infield, o = outfield, D’Amico: lr = low risk, ir = intermediate risk, ISUP = International Society of Urological Pathology

### Inclusion criteria and treatment planning

An interdisciplinary team of urologists and radiologists performed patient selection. Decision on suitability for TULSA was based on clinical parameters, histopathology from targeted and additional systematic biopsies, MRI findings, CT findings if applicable, findings from transrectal ultrasound and patient preferences.

Patients with histopathologically proven localized low and/or low-intermediate risk PCa were included (ISUP grade 1 /D’Amico low risk in n = 6, ISUP grade 2 /D’Amico intermediate risk in n = 14 and 2 patients with recurrence after previous radiotherapy). D’Amico low risk was defined as GS 3 + 3 = 6, PSA < 10 ng/ml, D’Amico intermediate risk was defined as GS 3 + 4 = 7a, PSA 10–20 ng/ml.; All patients refused radical prostatectomy or external beam radiation therapy (EBRT), and signed informed consent for TULSA as an individual treatment approach. Extent of tissue ablation was predefined based on results from prostate MRI and prostate biopsy. Patients with prostatic calcifications > 1 cm that were found on CT or ultrasound examinations were not treated with TULSA. Reliable heat application to achieve thermal coagulation (> 55°) of prostate tissue is specified with a radius of 2–3 cm from the urethral ultrasound applicator. Therefore, inclusion criterion for TULSA therapy was a prostate radius of ≤ 3 cm. The treatable volume was defined by histopathological results in combination with tumors visible on MRI.

### TULSA procedure

All TULSA procedures were performed in a 1.5 Tesla MRI (Siemens Aera, Siemens Healthineers, Erlangen, Germany). Patients were under general anesthesia during the entire procedure. Simultaneously to patient anesthesia induction, the MRI suite was prepared for TULSA treatment by experienced MRI technicians and radiologists. The urologist joined as soon as the patient entered the MRI suite and placed the Ultrasound applicator (UA) in the prostatic urethra as well as the rectal cooling device adjacent to the prostate. Both devices were perfused with degased water during heat application to protect the rectum and the urethra from thermal injury. The correct placement of both devices was verified in a sagittal T1-weighted sequence before the start of the intervention. The ultrasound probe used in the system is made of non-ferromagnetic materials such as plastic and ceramic, which are compatible with the strong magnetic fields used in MRI. The technique of TULSA treatment has been described before [4–6]. Briefly, a robotic arm holds the UA during the procedure and performs a rotational movement to apply heat to the specified regions of the adjacent prostate tissue. The UA consists of 10 single element ultrasound transducers that can be switched on and off separately. The depth of the ultrasound energy penetration during the TULSA procedure is influenced by the size and tissue characteristics of the prostate gland. Calcifications hinder ultrasound waves, potentially causing inadequate treatment, while cysts can enhance ultrasound waves, resulting in excessive treatment. Consequently, the depth of the ultrasound energy penetration can vary. Typically, the system can deliver heat up to 30 millimeters from UA to the periphery, and it has been utilized to treat prostates up to 250 cc in volume. Therefore, heat application can be planned individually for prostates up to 250 cc in volume. To ensure the safety of the internal sphincter we kept a safety margin between the sphincter and the first active transducer margin at the prostate apex. Anatomical MR images were acquired to plan and delineate the area in the prostate to be treated with TULSA on the treatment delivery console. These images were segmented by the radiologist in collaboration with the urologist. Main focus was an adequate coverage of the lesions visible in MR to the capsule of the prostate whilst avoiding damage to neurovascular bundles, the apical sphincter, the rectal wall, and the bladder neck.

Thermometry images were acquired before treatment as test images to exclude any signal disturbances and assure good image quality. The treatment delivery was then initiated under the guidance of continuous MR thermometry and temperature control in a closed loop. Additionally, we constantly monitored the patient’s movement. Based on real-time MR thermometry, the system automatically adjusted ultrasound power to prevent both under- and overheating. Contrast-enhanced MRI was performed after the ablation was terminated and non-enhancing prostate tissue was assumed as successfully treated tissue. Difficulties, complications, challenges and disturbances were documented.

### Follow-up

Follow-up visits were offered every 3–6 months after TULSA procedure for oncological and functional assessment. The Expanded Prostate Cancer Index Composite (EPIC) score was used to evaluate patients urinary, erectile, hormonal and bowel functions after TULSA treatment [8, 9]. Follow-up mpMRI was performed 6 months after treatment and MRI/ultrasound fusion-guided biopsy was performed after 6–12 months, as recommended for focal treatments according to experts’ consensus [10–12]. The biopsy technique comprised 3–8 targeted biopsy cores and additional 6–12 systematic biopsy cores.

### Ethics

Written informed consent was obtained from all patients in this study. Local Institutional Review Board approval was obtained (internal review board no.: 3530 − 2017).

### Statistical analysis

All statistical test were carried out using SPSS software version 27 (IBM). Kolmogorov-Smirnov tests were applied to test for normal distribution of data. Parametric (Analysis of variance (ANOVA), t-test) were chosen for normally distributed data and nonparametric tests (Friedman test, Wilcoxon rank sum test) were used for non-normally distributed data. A p-value < 0.05 was considered statistically significant. Values are given as mean ± standard error of the mean (SEM) if not indicated differently.

## Results

### Treatment, procedural and peri-procedural adverse events

**Fig. 1:**
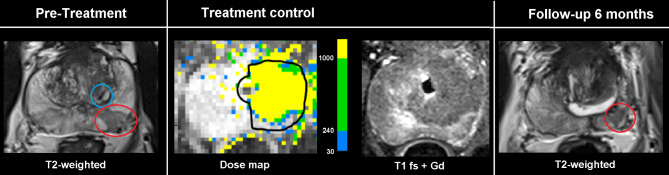
Calcification and treatment failure. Images of a 55-year-old patient with localized PCa in the left peripheral posterior zone (Gleason 3 + 4) undergoing a TULSA hemiablation. A small linear hypointense calcification is depicted in the pre-treatment T2-weighted images (blue circle). The tumor is marked by a red circle. A clear undertreatment distal from the calcification within the segmented tissue (black line) is visible in the dose map and viable tissue is shown in the T1-weighted image after contrast administration. The residual tumor is verified in the T2-weighted follow-up examination after 6 months (red circle)

**Fig. 2:**
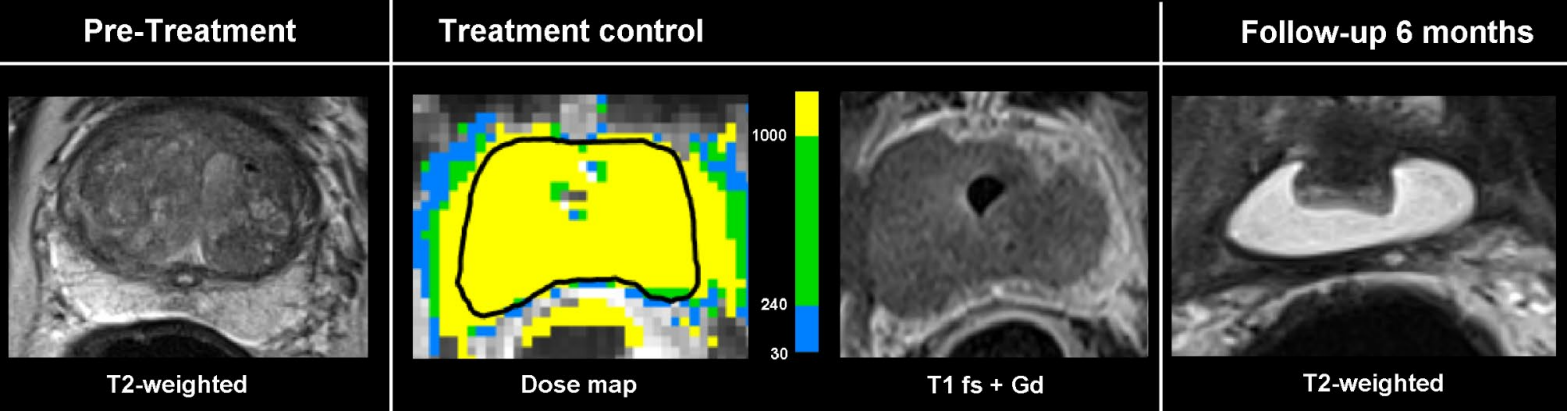
Viable tissue after ablation. Images of a 66-year-old patient with PCa in the peripheral posterolateral zone on the left (Gleason 3 + 3) and in the peripheral right apical zone (Gleason 3 + 4) undergoing TULSA whole-gland ablation. The tumor was proven via biopsy and could not be detected not demarked in the MR-images. Almost the entire segmented tissue reached the threshold for the calculated cell death in the dose map (green and yellow voxels). A small rim of enhancing tissue is visible in the left peripheral zone after treatment. Nevertheless, no residual viable prostatic tissue is detected in the T2-weighted follow-up examination after 6 months, and no recurrence has occurred to this day (19 months after treatment)

No acute complications were documented during TULSA treatment in 22 patients. In one patient with assumed calcification within the prostate tissue, MR-thermometry showed an artifact, probably due to acoustic shadowing and consecutive undertreatment. In this patient, viable prostate tissue distal from the artifact was found in post-procedural contrast-enhanced T1 images (Fig. 1). In this particular case, TULSA treatment was a salvage procedure after radiotherapy, and residual tumor after TULSA was found during follow-up. Interestingly, in a total of 6 patients, viable tissue, defined as contrast-enhancing tissue, was found after TULSA, even though thermometry showed full coverage during the heat application and no calcifications were detected (Fig. 2).

The mean duration for catheterization was 48 h. Catheters were removed on the second postoperative day, and patients were discharged after post-voiding ultrasound control. One patient developed an episode of urinary retention with the need of re-catherization at day 2 after procedure. Second removal under de-swelling therapy with NSAID was successful and voiding was possible. One patient presented with symptoms of pelvic pain that could be successfully treated with oral analgesia.

### Functional outcomes

**Fig. 3:**
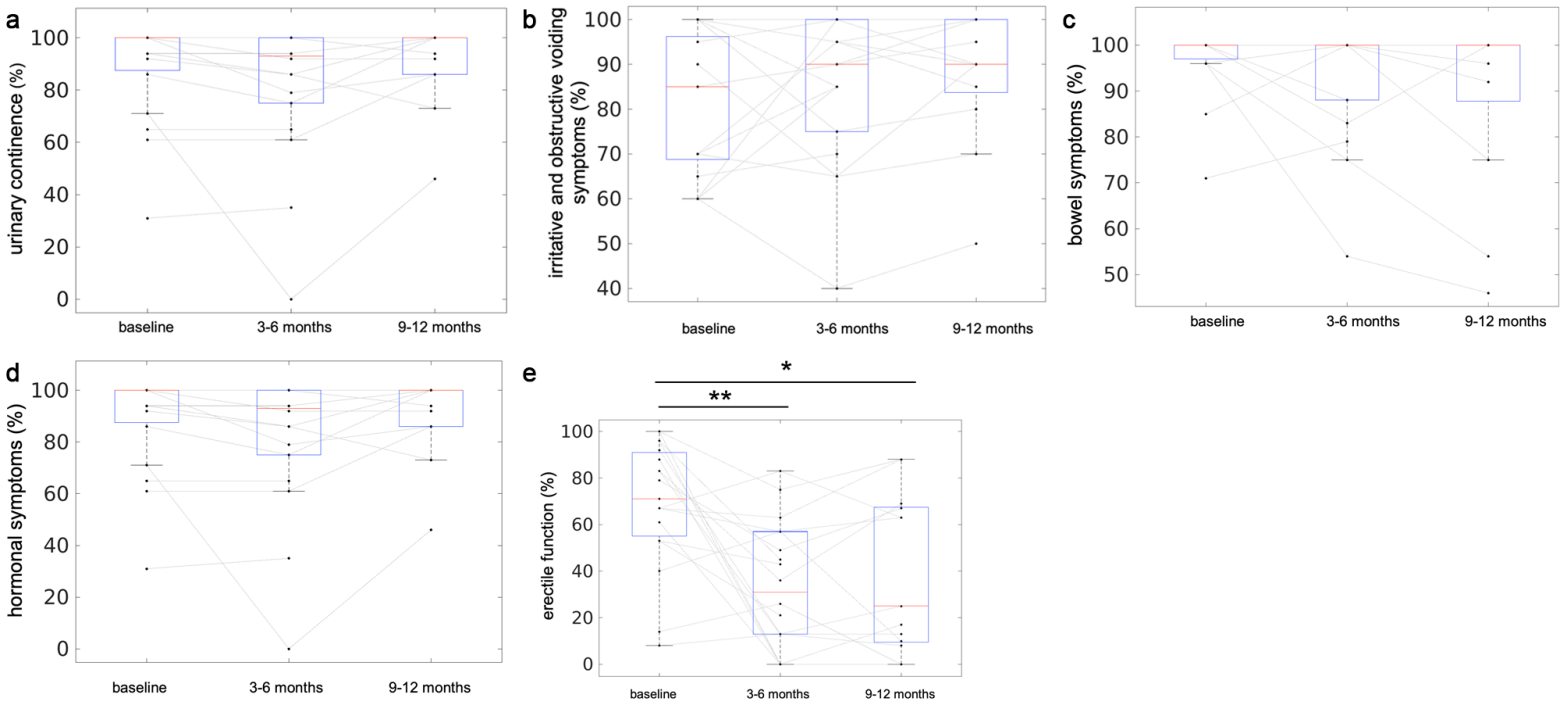
Functional assessment over a Follow-up period of 12 months using the Expanded Prostate Cancer Index Composite (EPIC) score. Depicted are results of functional assessment at baseline and up to 12 months after TULSA for urinary continence (**a**), irritative and obstructive voiding symptoms (**b**), bowel symptoms (**c**), hormonal symptoms (**d**) and erectile function (**e**). Red lines with blue boxes represent the group median with interquartile range for the corresponding data. Each black dot represents one patient at one time point and black dots of the same patient are connected with grey lines to present the individual development of functional parameters over time

Evaluation of functional parameters using the EPIC score was available for 19 patients before treatment (baseline), 17 patients after 3–6 months and for 11 patients after 9–12 months. Functional outcomes are visualized in Fig. 3. According to the results of the EPIC score, no significant changes over time were found for urinary continence (baseline: 89 ± 19%, 3–6 months: 86 ± 19%, 9–12 months: 90 ± 5%), irritative and obstructive voiding symptoms (baseline: 82 ± 4%, 3–6 months: 85 ± 4%, 9–12 months: 91 ± 3%), bowel symptoms (baseline: 97 ± 2%, 3–6 months: 93 ± 3%, 9–12 months: 93 ± 5%) and hormonal symptoms (baseline: 91 ± 3%, 3–6 months: 77 ± 5%, 9–12 months: 87 ± 4%, Fig. 3a-d). Erectile function significantly decreased compared to baseline (69 ± 6%) after 3–6 months (31 ± 6%, p < 0.01) and stayed impaired until 9–12 months after TULSA treatment (32 ± 10%, p < 0.05, Fig. 3e).

### Laboratory outcomes

**Fig. 4:**
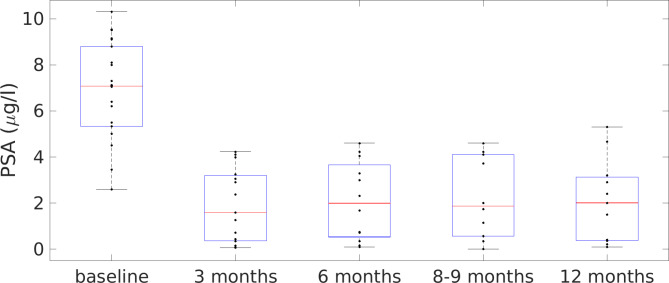
PSA-values over the follow-up period of 12 months after TULSA. Developments of PSA values during a follow-up period of 12 months are visualized. PSA significantly decreased after TULSA.

Mean PSA values after TULSA (measured every 3 months during follow-up) were significantly lower compared to pre-surgical PSA values (mean ± standard deviation: 2.1 ± 1.8 vs. 6.8 ± 2.1 ng/ml, p < 0.001). Developments of PSA values during a follow-up period of 12 months are visualized in Fig. 4.

### Imaging and histopathology

Multiparametric prostate MRI was performed after 6 months in 21/22 patients. One patient refused MRI; in this patient PSA values declined from 9.5 ng/ml to 0.1 ng/ml, we therefore did not suspect recurrence. Out of 21 prostate MRI examinations, 15 did not show any signs of recurrent or residual PCa. The Prostate Imaging Reporting and Data System (PIRADS) score is not designed for the classification of lesions that appear in pre-treated prostates, therefore, the PIRADS score in not applicable for the follow-up MRI examinations in our patient collective. In order to make interdisciplinary communication easier, we still applied PIRADS criteria for the interpretation of MRI examinations for the likelihood of the presence of clinically significant cancer with PIRADS 1 = (very low) clinically significant cancer is highly unlikely to be present, PIRADS 2 = (low) clinically significant cancer is unlikely to be present PIRADS 3 = (intermediate) the presence of clinically significant cancer is equivocal, PIRADS 4 = (high) clinically significant cancer is likely to be present, PIRADS 5 = (very high) clinically significant cancer is highly likely to be present.

In 4/22 MRI examinations, clinically significant PCa was likely to be present (PIRADS 4). In 2/22 MRI examinations, the presence of clinically significant cancer was equivocal (PIRADS 3).

Histopathology from MRI/ultrasound fusion-guided biopsy was available in 14/22 cases. Biopsy was not performed in 8 cases; 7 of these 8 patients received prostate MRI in which clinically significant cancer was unlikely to be present (PIRADS 2) and PSA values significantly decreased compared to pre-surgical values (1.8 ± 1.5 ng/ml vs. 6.4 ± 2.3 ng/ml, p < 0.05), so they refused biopsy upon these clinical results. One patient received PSMA-PET-CT due to a consistency in PSA elevation and presented then with lymph-node metastasis. Of note, this patient was likely undergraded in his primary diagnosis.

In 3 out of 14 patients receiving a control biopsy due to PIRADS 4 results on mpMRI, GS 3 + 4 = 7a PCa was found, 1 patient with outfield recurrence and in 2 cases with in- and outfield recurrence. In one patient (presenting with PIRADS 3 in the follow-up MRI), Gleason 4 + 3 = 7b was found (outfield recurrence). One patient with PIRADS 3 presented with bone metastases, but no intraprostatic PCa was found in MRI/ultrasound fusion-guided biopsy. Of note, in this patient we performed a salvage TULSA treatment due to recurrent PCa disease after EBRT. Since no PCa was found in histopathology of MRI/ultrasound fusion-guided biopsy we assumed that local tumor control might have been successful, so initial diagnosis of localized PCa disease was possibly incorrect. Therefore, we excluded this patient from calculation of recurrence rate. The overall intra-prostatic recurrence rate in our study collective, when considering only patients without prior treatment, amounts to 20% (4/20 patients), and 10% for infield recurrences (cancer that occurred in the treated tissue). Patients’ characteristics and follow-up are summarized in Table 1.

## Discussion

TULSA treatment of localized prostate cancer was safe and effective in our patient collective of 22 men with six whole gland and 16 focal ablations. No major adverse events or complications in the post-operative period were documented. Nevertheless, we could observe a decrease of erectile function in our small cohort. No changes in urinary continence, voiding symptoms, bowel irritation or hormonal symptoms were reported.

Urinary retention as a perioperative complication has previously been reported by one study group after whole-gland TULSA [3], but not after focal TULSA [5]. In our study, urinary retention was documented in only 1 out of 22 patients after focal ablation.

In the follow up period of 12 months, no significant changes concerning urinary continence or irritative/obstructive voiding symptoms, bowel irritations or hormonal symptoms were found. This finding is in line with previous data; in a large, multicenter prospective study, whole-gland TULSA had a low risk of functional decline: no bowel irritation or injury was documented and 96% of men returned to baseline urinary continence [4]. Other studies also reported that urinary urgency improved - particularly in patients with benign prostate hyperplasia − 12 months after TULSA treatment due to a downsizing effect [13, 14].

One of the advantages of TULSA compared to transrectally applied high-intensity focused ultrasound (HIFU) is that heat application is transduced from the urethra, with the rectum being protected using an intrarectal cooling system. In line with this, bowel problems after TULSA occurred neither in our study nor in previously published data [4, 6, 13, 15]. Of note, in our cohort erectile function significantly declined after 3–6 months (p < 0.01) and after 9–12 months (p < 0.05) compared to baseline. Results on erectile function after TULSA from previous studies are very heterogenous; Klotz et al. reported a significant decline of erectile functional and overall sexual function compared to baseline at 1, 3, 6 and 12 months after TULSA from their multicenter study including data of 115 patients [4]. Nevertheless, in the same study, 75% of the subgroup of men who were potent at baseline maintained or returned to baseline erectile function. Nair et al. present quality of life parameters of 22 men 3 years after TULSA with no significant difference in erectile function found after 3 years. It has to be noticed that 10 out of 22 men had a remarkable decrease in erectile function, while 5/22 had a clinically important improvement [16]. In our study collective, no improvements of erectile dysfunction compared to baseline were found, therefore, the mean value for the sexual domain of the EPIC score remained significantly lower. However, Fig. 3e suggests (similarly to the other studies), a gradual recovery of erectile function after 12 months compared to 6 months (even though it is still significantly impaired after 12 months compared to baseline). Eltermann et al. and Antinnen et al. report no changes in erectile function 12 months after TULSA in 9 men with BPH and concurrent PCa [14] and 11 men after salvage TULSA [17]. In both of these studies, preTULSA erectile function was decreased with an IIEF baseline of 14.6 [14] and an EPIC of 18% [17], respectivly. Mean baseline EPIC score in our study was 69%, therefore, results of the studies are not comparable.

Further research is necessary to identify patients at risk for loss of erectile function after TULSA. Recent studies demonstrate that erectile dysfunction imposes not only a substantial quality of life burden on men but also a significant economic burden for their employers [18–20] and patients might choose quality of life over cancer specific survival [21]. Knowlegde on possible side effects of focal treatment are therefore essential in order to be able to make informed decisions on treatment options. In our study cohort, we observed a significant impairment in sexual function, even more than in other studies reported. Of course, we are limited due to the small number of cases. But our data corroborate the need for a better stratification and particularly the need for an elaborate discussion with the patient.

In our patient collective, intraprostatic residual/recurrent PCa was diagnosed in 5/22 patients (23%) within the first 6–12 months after TULSA. One patient presented with bone metastasis while no intraprostatic PCa was found.

Recurrence rates are in line with results from other study groups [4]. For example, 9/29 patients had clinically significant PCa and 7/29 clinically insignificant PCa 12 months after whole-gland TULSA, which results in a recurrence rate of 56% in total [16, 22]. Interestingly, in this study, all patients negative for PCa at 12 months remained negative after 3 years. So, first biopsy at 12 months after treatment could possibly serve as a surrogate for long lasting treatment success, emphasizing the need of control biopsy after focal treatment approaches. In another recent study, reporting whole-gland as well as focal TULSA treatment, a recurrence rate of 14/52 patients (27%) is described [6]. Main reasons for recurrence in this study were insufficient thermal margins around the tumor and calcifications disrupting the beam path [6].

In one of our patients, intraprostatic calcification led to acoustic shadowing of the ultrasound waves and therefore heat could not be applied in these areas of the prostate, and residual tumor was found in the follow-up biopsy (Fig. 1). Intraprostatic calcifications have been reported to be problematic for TULSA before and are a clear limitation for TULSA treatment [4, 6]. Klotz et al. reported from the prospective multicenter trial that intraprostatic calcifications at screening were a predictor for persistent grade group 2 PCa at 12 months [4]. The suggestion was that patients with calcifications greater than 1 cm are excluded from TULSA treatment. However, Klotz et al. reported that calcifications smaller than 1 cm also caused acoustic shadowing and were associated with a higher risk of residual PCa [4]. We therefore subsequently adapted selection criteria for TULSA treatment and screened for intraprostatic calcifications by adding transrectal ultrasound to the pre-treatment examinations excluding patients with calcifications.

Residual and/or recurrent disease raises the question about salvage treatment options and its feasibility with respect to functional and oncological outcomes; several studies concentrating on this very important issue are currently carried out.

Nair et al. report their experience with salvage open radical prostatectomy after TULSA treatment in four patients with PCa recurrence [23]. In this study, radical prostatectomy was a save option after TULSA and periprostatic TULSA side effects did not lead to increased perioperative complications. Moreover, Lumiani and colleagues repeated TULSA treatment in case of recurrence disease (n = 9) and could demonstrate disease control in 89% of patients. This indicates salvage treatments to be an option in case TULSA fails.

Astonishingly, even though therapy planning included the prostate tissue from the urethra to the organ capsule and thermometry showed full coverage during the heat application, we still found contrast-enhancing tissue in the treated area in 6 patients (27%) directly after TULSA. An example is given in Fig. 2; in the post-contrast control images, the non-enhancing tissue is smaller than planned and expected from thermometry and remaining viable prostate tissue in the posterior peripheral zone on the left side of the prostate must be assumed. However, recent data suggests that the non-perfused volume directly after TULSA treatment underestimates the ablation zone measured after 12 months by up to 50% [22]. Therefore, the perfused volume seen after treatment might not correspond to viable tissue after 12 months. Contrary to this, in another study evaluating whole-gland TULSA, a safety margin of 3 mm, equivalent to 10% of the peripheral zone, was left in order to preserve healthy tissue [3]. In this mentioned study, clinically significant PCa was found in 10/29 patients (35%) and cancer of any stage was detected in 17/29 patients (59%) following almost whole-gland TULSA.

In our study, 4/6 patients showing viable tissue directly after TULSA had recurrent or residual disease in the follow-up histopathology, however, the number of patients is too small to draw general conclusions from this finding.

Nevertheless, the intention of focal therapy is to identify and treat the index lesion. The index lesion, defined as the largest lesion in the prostate with the highest Gleason grade, is assumed to drive disease progression and focal ablation could therefore enable PCa control while preserving quality of life [24]. Therefore, a small rim of contrast-enhancing prostate tissue after TULSA might be an acceptable finding, given that the index-lesion is properly treated. Furthermore, in a recent study that evaluated choices of men with low-intermediate (n = 468) and high-risk (n = 166) PCa, patients preferred quality of life over cancer specific survival [21]. Considering this, treatment of the index lesion with heat application in a nerve-sparing manner minimize side-effects might be desirable.

Our study has limitations, mainly due to the study design. Firstly, the number of patients is relatively small and no subgroup analysis was performed due to small subgroups from which conclusions cannot be drawn. Secondly, in 8 patients no MRI/ultrasound guided biopsies were performed. As described by other authors, routine biopsy after focal therapy is refused by many patients [6]. These patients were monitored by MRI examinations and PSA measurements as suggested before [25]. Nevertheless, it has been reported that after focal ablation, MRI of the prostate has a negative predictive value of > 90% for clinically significant recurrence [26, 27]. Thirdly, TULSA was performed on a 1.5 Tesla MRI in our study, while previous studies used a 3 Tesla MRI. Therefore, thermometry data might differ limiting comparability of data. However, the thermometry is sufficiently accurate at 1.5T, making treatment possible with at this magnetic field strength [28].

In conclusion, establishment of the system into clinical routine was unproblematic and short-term outcome seems to be encouraging. Primary focal-ablative treatment was associated with a total of 4 patients suffering from recurrence: 20% infield and outfield; 10% of patients presented with infield recurrence only. In three patients the tumor was defined as low-intermediate – ISUP II risk group.

Impairment in erectile function can be observed, underlining the need for an elaborate discussion with the patient.

## Data Availability

The datasets used and/or analysed during the current study available from the corresponding author on reasonable request.

## Abbreviations

ANOVA: Analysis of variance
EBRT: External beam radiation therapy
EPIC: Expanded Prostate Cancer Index Composite
GS: Gleason score
HIFU: High intensity focused ultrasound
ISUP: International Society of Urological Pathology
mpMRI: Multiparametric magnetic resonance imaging
PCa: Prostate cancer
PIRADS: Prostate Imaging and Reporting Archiving Data System
TOOKAD: Vascular-targeted photodynamic therapy
TULSA: Transurethral ultrasound ablation
UA: Ultrasound applicator
